# Frequency and clinical patterns of stroke in Iran - Systematic and critical review

**DOI:** 10.1186/1471-2377-10-72

**Published:** 2010-08-23

**Authors:** Akram A Hosseini, Davood Sobhani-Rad, Kavian Ghandehari, Hani TS Benamer

**Affiliations:** 1 Neurology Department, University Hospital Coventry and Warwickshire, Coventry, UK; 2 Paramedical Faculty, Mashhad University of Medical Sciences, Mashhad, Iran; 3 Neurology Department, Mashhad University of Medical Sciences, Mashhad, Iran; 4 Neurology Department, Royal Wolverhampton Hospital, Wolverhampton, UK

## Abstract

**Background:**

Cerebrovascular disease is the second commonest cause of death, and over a third of stroke deaths occur in developing countries. To fulfil the current gap on data, this systematic review is focused on the frequency of stroke, risk factors, stroke types and mortality in Iran.

**Methods:**

Thirteen relevant articles were identified by keyword searching of PubMed, Iranmedex, Iranian University index Libraries and the official national data on burden of diseases.

**Results:**

The publication dates ranged from 1990 to 2008. The annual stroke incidence of various ages ranged from 23 to 103 per 100,000 population. This is comparable to the figures from Arab Countries, higher than sub-Saharan Africa, but lower than developed countries, India, the Caribbean, Latin America, and China. Similarly to other countries, ischaemic stroke was the commonest subtype. Likewise, the most common related risk factor is hypertension in adults, but cardiac causes in young stroke. The 28-day case fatality rate is reported at 19-31%.

**Conclusions:**

Data on the epidemiology of stroke, its pattern and risk factors from Iran is scarce, but the available data highlights relatively low incidence of stroke. This may reflect a similarity towards the neighbouring nations, and a contrast with the West.

## Background

**Figure 1:**
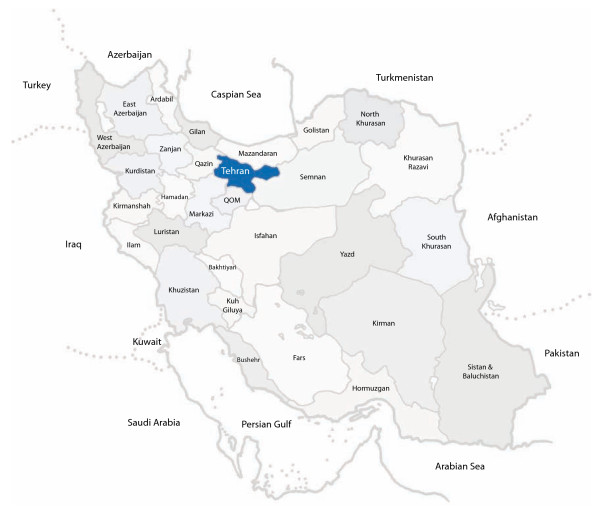
**Map of Iran**.

Iran, known as Persia until 1935, is located in Southwest Asia (figure 1). With an area of 1,648,000 square kilometers, Iran ranks sixteenth in size among the countries of the world and its climate ranges from subtropical to subpolar[1]. Its has a population of 70 million, with more than 13 millions living in the capital Tehran[2]. One quarter of its people are 15 years of age or younger, compared with 7.26% of the population aged 60 or over[2]. Iran with its Indo-European origin and historically being a major crossroad for human migration is a country composed of different ethnic groups including Persian (51%), Azeri (24%), Gilaki and Mazandarani (8%), Kurd (7%), Arab (3%) as well as, Baluch, Turkman, Jews, Armenians, Assyrians and Zoarastians[1].

Over the last couple of decades, there has been major improvement in education and health services. The number of universities has grown from 22 in 1978 to 54 state universities and 289 major private universities in 2009[3]. The Iranian Medical Council reports over 100,000 physicians, including 660 neurologists, practising in the year 2009[4]. However, about 150 neurologists are based in Tehran with over 40 MRI scanning centres, at least double than the necessary epuipment for the population[5]. According to the World Health Organisation (WHO), life expectancy at birth in Iran is estimated at 69 years for male and 73 years for female in 2006[6]. The total expenditure on health in 2005 is covered by the government and the private sector at 55.8% and 44.2%, respectively[6].

Cerebrovascular disease is the second commonest cause of death, and the 6^th ^leading cause of diseases burden globally and expected to move to the 4^th ^place by 2020[7]. Over 80% of all stroke deaths in the world occur in the developing countries[8]. However, stroke is largely preventable, so knowledge of risk factors within a certain country is an essential step in reducing the stroke rate and resulting disease burden. Also, lifestyle and vascular risk factors such as hypertension and diabetes vary among different countries and cultures. An examination of stroke frequency and clinical pattern in various nations is therefore important to understand the pattern of the disease in a certain population with the ultimate aim of reducing the stroke rate. Effective prevention programmes should also be based on data relevant to the country under study. The literature representing the burden of stroke in developing countries and Asia is lacking comprehensive data on Iran. Epidemiological studies of stroke in these countries are constantly bound to numerous limitations such as inadequate nationwide data sets, lack of standard population-based studies, flawed medical registration and discontinuity of data maintenance between the family physicians, state healthcare system as well as private sector. Furthermore, those published, can remain inaccessible when written in the local or national Persian journals. The aim of this paper is to systematically review the data related to the frequency, risk factors, types and mortality of stroke in Iran. This should identify future potential research areas and help in medical service planning in the Iran. To our knowledge, no previous systematic reviews of stroke in Iran have been undertaken.

## Methods

### Data collection

The data was collected over four stages: -

#### Stage 1 - search through PubMed

Publications on Stroke in Iran were identified by searching the PubMed using the keywords:- "stroke", "intracranial bleed", "intracranial haemorrhage", "brain infarction", "cerebrovascular disorders", "cerebrovascular disease", "epidemiology", "incidence", "prevalence" combined with "Iran", "Iranian", "Persian", "Persia" and "Farsi".

Titles and abstracts were scanned by one author (AH) to identify eligible studies according to agreed inclusion and exclusion criteria. The full paper of potential studies was retrieved for more detailed assessment.

#### Stage 2 - search through "Iranmedex" and Iranian University Index Libraries

IranMedex is the website for Iranian medical index database (http://www.iranmedex.com). The website is a search engine to identify studies published in Persian or English, including articles in Persian or International journals, medical thesis or scientific reports. IranMedex enables one to purchase some articles or abstracts but not all.

The above mentioned keywords were used to identify articles by searching Iranmedex for further articles. The titles of all related articles and medical theses were reviewed. Since the full papers of many studies were not available in the website of "Iranmedex", one author (DSR) searched medical index for each major medical school in Iran, and the well-known Iranian medical journals to get hold of the relevant papers. To access the medical theses, medical index libraries were visited to copy or scan the relevant parts and forward it to the co-author (AH).

#### Stage 3 - search the references of the relevant papers

Each reference in all relevant papers was checked for any additional articles.

#### Stage 4 - Official Data on "Burden of Disease in Iran"

The data for the year 2003 is published in a book "National Burden of Disease & Injury in Iran", so-called "The Green Book", with exclusive rights to the Ministry of Health. It reports the results of the study on the national burden of disease. The ongoing project is implemented by the Ministry of Health and Medical Education including a team of epidemiologists, sociologists, medical and dental specialists. The study is conducted in 6 provinces including: Eastern Azarbayejan, Booshehr, Chahar Mahal & Bakhtiari, Khorasan, Hormozgan and Yazd. A software coding system, which is comparable with global burden of disease studies, is designed for the study to collect the data from both hospitals and primary health care systems.

The access to the official data was obtained via direct communication with the current Ministry of Health in Iran. Although the data has valuable information on the nationwide burden of diseases, it does not carry strong pathophysiological and epidemiological value[9]. The reported incidence and mortality statistics are limited by inaccuracy, variable diagnostic and coding methods by both medical and non-medical administrative staff, and the absence access to detailed information on the cases, pathology subtypes, methods and individual studies. We therefore, did not include the data in this study.

### Inclusion and exclusion criteria

Papers were selected for this systematic review if they fulfilled the following criteria:

1. The study was conducted in Iran,

2. The stroke is clearly defined, the definition varied in different studies: mostly defined as a focal neurological deficit, confirmed by a trained physician, that persisted for at least 24 hours; all the cases recruited to the study had brain computed tomography (CT) or magnetic resonance imaging (MRI) and underwent batteries of standard investigations including at least electrocardiography, blood count, serum electrolytes, blood sugar and lipid profile.

3. The study contained data about frequency (incidence, prevalence) or clinical pattern (types, risk factors and outcome) or mortality rate of stroke,

4. The study was published before 2^nd ^February 2009, and written in English or Persian,

5. Studies on cerebral venous sinus thrombosis and comparative studies on individual stroke risk factor analysis were excluded.

### Data extraction

The following data were extracted from identified papers: stroke incidence, prevalence, patient sex and age, stroke type, risk factors, and clinical outcome. The included articles were reviewed and data was analysed descriptively. Statistical analysis or meta-analysis was not attempted due to significant variability in both data sources and study methodologies.

## Results

**Figure 2:**
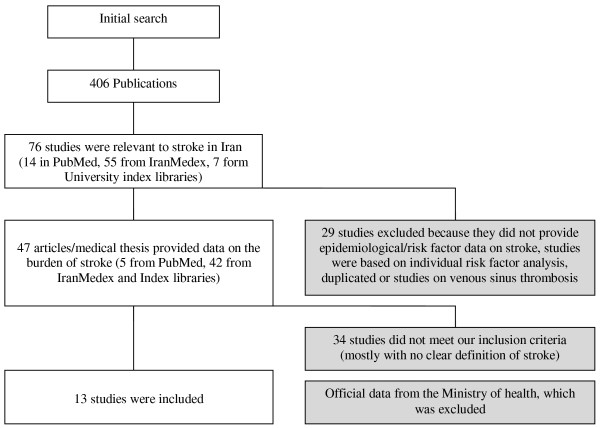
**Details of data selection in this review**.

The data selection process is shown in figure 2. The details of all the stroke studies in Iran, both included and excluded from this study, are tabulated in table 1. Thirteen articles, which fulfilled the inclusion and exclusion criteria, are included in this systematic review[10-22].

**Table 1:** Summaries of all studies on stroke in Iran

Study	City or province	Study period in years	Published Language	Included for assessment
Pakdaman[51]	Tehran	Four, 1986-1990	Persian	No
Ashrafi[52]	Urmia	Two, 1991-1992	Persian	No
Janghorbani, et al[53]	Kerman	Two, 1992-1994	English	No
Mahmoodi[54]	Yazd	< two, 1993-1994	Persian	No
Khandaghi[55]	Tabriz	One, 1994	Persian	No^a^
Azimian, et al[56]	Hamadan	One, 1994-1995	Persian	No
Masoud, et al[57]	Kashan	One, 1996-1997	Persian	No
Ashrafi, et al[58]	Urmia	One,1997 (1 yr)	Persian	No
Miabi, et al[59]	Tabriz	Three, 1996-1999	Persian	No
Ravari, et al[60]	Rafsanjan	Three, 1996-1999	Persian	No
Noor-Mohammadi, et al[61]	Gilan	Two, 1996-1998	Persian	No
Harirchian, et al[62]	Tehran	Five, 1997-2002	English	No
Andelib, et al[63]	Bushehr	< half a year, 1998	Persian	No
Asadpour, et al[64]	Sanandaj	One, 1998	Persian	No
Masoud[65]	Tehran	< a year, 1998	English	No
Ebrahimi, et al[23]	Kerman	One 1998-1999	English	No
Razaazian, et al[66]	Kermanshah	Two,1998-2000	Persian	No
Hosseini, et al[67]	Gorgan	One, 1999	Persian	No
EmadZadeh, et al[68]	Mashhad	Three, 1999-2002	Persian	No
Togha, et al[22]	Tehran	Three, 1999-2002	English	Yes^a^
Sabet, et al[69]	Isfahan	Two, 2000-2002	English	No
Oveisgharan, et al[20]	Isfahan	Three, 2000-2003	English	Yes
Mousavi, et al[46]	Isfahan	Five, 2000-2004	English	No
Ghandehari, et al[70]	South. Khorasan	Five, 2000-2005	English	Duplicate
Ghandehari, et al[15]	South. Khorasan	Five, 2000-2005	English	Yes
Nikseresht, et al[19]	Shiraz	Three, 2001-2003	Persian	Yes
Ahangar, et al[10]	Babol	Two, 2001-2003	English	Yes
Ahangar, et al[71]	Babol	Two, 2001-2003	Persian	Duplicate
Ghandehari, et al[17]	South. Khorasan	Five, 2001-2005	English	Yes
Ghandehari, et al[72]	South. Khorasan	Five, 2001-2005	English	Duplicate
Savadi Oskoui, et al[73]	Ardabil	Half a year, 2002	Persian	No
Savadi Oskoui, et al[74]	Ardabil	Half a year, 2002	Persian	No
Noubahar, et al[75]	Semnan	One, 2002-2003	Persian	No
Sarsar Shahi, et al[24]	Urmia	Four, 2002-2006	Persian	No^a^
Ghandehari, et al[12]	South. Khorasan	Five, 2002-2007	English	Yes^b^
Parniya, et al[21]	Ardabil	One, 2003	Persian	Yes^a^
Dodangeh, et al[76]	Tehran	< half a year 2003	Persian	No
Tavassoli, et al[77]	Tehran	Two, 2003-2005	Persian	No^b^
Amini Sani, et al[78]	Ardabil	< two, 2004-2005	Persian	No^a^
Hashemilar, et al[79]	Ardabil	One, 2004-2005	Persian	No
Ghandehari, et al[16]	South. Khorasan	One, 2005-2006	English	Yes^a^
Ghandehari, et al[11]	South. Khorasan	One, 2006	English	Yes^a^
Ghandehari, et al[14]	South. Khorasan	One, 2006-2007	Persian	Yes^a^
Ghandehari, et al[18]	Mashhad	One, 2007	English	Yes^a^
Ghandehari, et al[13]	Mashhad	Two, 2006-2007	English	Yes^a^
Iranmanesh, et al[80]	Rafsanjan	Not clear	English	No
Iranmanesh, et al[81]	Rafsanjan	Not clear	Persian	No

^a^The study does not report incidence data, but designed for risk factor, mortality or prognosis analysis. ^b^Study on Paediatric cases.

### Incidence

There is only one study showing an incidence of 43.12/100,000 population/year of first-ever ischaemic stroke in all age range in Iran[17]. Whilst a study showed an annual incidence rate of 22.7 per 100,000 population for first ever and recurrent stroke, another reported a rising incidence from 84.16 to 103.23/100,000 population over four years[10,20]. Ischaemic stroke events made up the majority in both the studies at 67% to 68% of the cases [10,20]. The annual incidence of young stroke (age 25-45) was 8 and 1.83/100,000 population for children below age of 15[12,15]. Non-traumatic brain haemorrhage was reported at 15.5 per 100,000 per year[23]. Summary of all incidence studies is shown in table 2.

**Table 2:** Stroke demographic data in Iran

Study	City or Province	Duration of collection	Sample population/number of events	Sex		Age range in years(mean in years)	Diagnostic criteria/CT or MRI	Case ascertainment	Study design	Event type	Total population per 100,000 (95% CI)
				**Male**	**Female**						
**Ahangar et al**[10]	Babol	2001 - 2003	550,000/250	120 (48%)	130 (52%)	All (68)	WHO/all	Hospital admission (the only centre for stroke admission in the region)	Retrospective	First-ever & recurrent	22.7
**Oveisgharan et al**[20]	Isfahan	2000 - 2003	1,700,000/4,361	2121 (49%)	2240 (51%)	All (68)	WHO/90%	Admission in 8 hospitals in the region (excluded 2 military hospitals)	Prospective	First-ever & recurrent	^a^84.16 (78.46-89.86) ^b^94.84 (88.94-100.74) ^c^100.18 (94.40-105.96) ^d^103.23 (97.41-109.05)
**^e^Ghandehari et al**[17]	Southern Khorasan	2001 - 2005	682,000/1,392	654 (47%)	738 (53%)	All (65.6)	^f^PIC/all	Hospital admission (the only neurology centre in the province)	Prospective stroke registry	First-ever	43.17
**^e^Ghandehari et al**[12]	Southern Khorasan	2002 - 2007	196,000/17	10 (59%)	7 (41%)	< 15 (5.5)	^g^Clinical definition/all	Hospital admission (the only neurology centre in the province)	Prospective	First-ever	1.83
**^e^Ghandehari et al**[15]	Southern Khorasan	2000-2005	314,000/124	64 (52%)	60 (48%)	15-45 (35.7)	^g^Clinical definition/all	Hospital-based study(the only neurology centre in the province)	stroke registry	First-ever	8

CT: Computerised tomography; MRI: Magnetic Resonance Imaging; CI: 95% confidence intervals stated when available; WHO: World Health Organisation. ^a^Incidence rate in 2000; ^b^Incidence rate in 2001; ^c^Incidence rate in 2002; ^d^Incidence rate in 2003; ^e^Only ischaemic strokes were included; ^f^PIC: Practical Iranian Criteria classification for aetiologic and topographic diagnosis of brain infarction; ^g^Stroke was defined as an ischaemic focal neurological deficit that persisted at least for 24 hours.

### Sex and Age

Stroke was reported to be slightly more common in females (range form 51%-53%) in the studies that included all age range[10,17,20]. However, stroke was more common in young males, age between 15-45, (52%)[15] and in boys (59%)[12]. The clinical series showed a mean age of stroke within the 7^th ^decade[10,17,20]. However, haemorrhagic stroke was reported more commonly in women (57.9% for intracranial haemorrhage)[21,24].

### Types of stroke

Only two studies reported all types of strokes[10,20]. Ischaemic stroke was reported in 67.2-68.45% of all stroke patients while primary intracerebral haemorrhage in 23.9-28.4% and subarachnoid haemorrhage in 2.9-4.4%[10,20]. One study reported that 36% of patients with ischaemic stroke had thrombotic event and 31.2% had an embolic one[10]. Another study showed that 64% of patients with ischaemic stroke had a territorial infarct, 19.5% a small deep infarct and 4.6% a border zone territory infarct[17]. In this study, the distribution of stroke with atherosclerotic and cardioembolic mechanism did not show significant difference between carotid versus vertebrobasilar territory involvement[17]. One study showed that vertebro-basilar territory infarctions in 17% of women and 20% of men presented with brain infarction[17]. The only study which registered "posterior circulation strokes", reported similar gender distribution, with brain stem being the most common site of stroke (59%) in the study group, followed by cerebellum (47%) and mixed topographies (16%)[13].

### Risk factors

Hypertension was the most frequent risk factor in both ischaemic stroke (54% of patients)[10,17] as well as spontaneous brain haemorrhage (70%-73.2% of cases)[21,23]. Cardiac causes (54%), mainly rheumatic heart disease (34%) were the most frequent risk factor in young stroke[15]. In adults over 35-years old, 20% of patients with ischaemic stroke had cardiac sources of embolism mainly rheumatic mitral stenosis[16]. In an aetiologic study on lacunar infarcts, hypertension, followed by diabetes and hypercholoestrolaemia were reported more frequently when compared with large vessel territory infarct. However, atrial fibrillation, ipsilateral carotid stenosis was more common in the latter; smoking and history of previous TIA were not significantly different between the two groups[11]. Summary of all risk factors of stroke in Iran are shown in table 3.

**Table 3:** Frequency of risk factors in cases of stroke admitted to hospital

Study	Risk factors											
	Event type	HBP (%)	DM (%)	Dyslipidaemia (%)	Cardiac diseases				Smoking (%)	CVD (%)	FH (%)	†Others (%)
					IHD (%)	AF (%)	RHD (%)	Not specified (%)	-	32.8	-	-
**Ahangar, et al**[10]	Stroke	54	24	26	19.2	16	-	8	26	-	-	-
**Ghandehari, et al**[17]	Stroke	53.2	13.5	8	12.2	11.4	17.7	2	15.2	22.3	10.6	†32.1
**Parniya et al**[21]	Intracranial Haemorrhage	36.6	5.1	2.55	-	-	-	6.95	9.55	-	-	-
**Ghandehari, et al**[11]	Lacunar infarct	60.4	20	23	-	3	-	23	14	15	-	‡27

HBP:Hypertension; DM: Diabetes mellitus; CVD: Previous history of cerebrovascular disease; FH: family history of stroke; IHD: Ischaemic heart disease; AF: Atrial fibrillation; RHD: Rheumatic heart disease. †Others risk factors reported include: low mobility, obesity and oral contraceptive. ‡ICS: Internal carotid artery stenosis

The study on the aetiologies of posterior circulation stroke, using Practical Iranian Classification[25], reported atherosclerosis being the most common cause in 50.6% of the cases, followed by uncertain causes (25.5%), cardioembolism (12.5%), combination of atherosclerosis and cardioembolism (6.3%) and miscellaneous causes (4.6%)[13].

### Outcome of stroke

The case fatality rate at 28 days for all type of stroke was 19.2% in one study[10], 31.5% in another[20]. Case fatality rate was low in ischaemic stroke (14-26%) in comparison with haemorrhagic stroke (37.6-68%)[10,20-22]. One study showed a mortality rate of 32% during a two year study period[10]. One hospital-based study reported 6.1% rate of vascular dementia subsequent ischaemic strokes, of those, 65% had lacunar infarct in isolation or association with large infarcts[14].

## Discussion

### Incidence

The available data suggest that the unadjusted incidence of stroke in Iran is comparable to the figures from Arab Countries[26-32], higher than sub-Saharan Africa[33], but lower than developed countries[34], India[35], the Caribbean & Latin America[36], and China[37]. The apparently lower incidence may be explained by the methodological artefact, country's relative young population and hospital-based case registry in the studies. The population aged over 65 in Iran is estimated at around 5%[2], compared with 16% and 13.6% over-65 age group in the United Kingdom and the United States of America, respectively[38,39]. This may have an impact on incidence reports in Iran as they are not age-adjusted. The similarities to stroke features from Arab countries might be related to ethnically heterogeneous population in Iran, consists of intermixed Persian-Arab culture, which was created since the invasion of Arabs in the 6^th ^century. Genetic susceptibility for stroke and risk factors are as a result of inter-marriage, co-evolution and free mixing between the individuals of both ethnic backgrounds, the impact of similar geography and environment, look-alike lifestyle and comparable relatively young population in the Arab nations may have played roles in the emergence of similar stroke pattern between Iran and Arab countries.

There has been one attempt at a community-based incidence study by running a stroke registry in the province of Southern Khorasan with over 680,000 habitants[2]. All possible stroke cases were to be referred to the Acute Stroke Unit for evaluation prior to discharge or admission. However, this has a significant impact on the data collection as it may exclude patients with mild disease or those who elect not to seek medical advice leading to under-reporting and artificially low incidence. Almost half the population seek medical advice from the private sector [6]. This figure decreases in hospital admissions but remains missing from the nationwide data particularly in the Capital Tehran with numerous private hospitals. Further studies with accepted quality criteria and age correction are essential to represent true incidence of the relatively young population of Iran.

None of the incidence studies fulfils criteria suggested by Sudlow and Warlow[40] and the updated version by Feigin[41], largely through missing a clear definition of stroke and methodological defect, in addition to lack of community-based case ascertainment, absence of age-adjusted incidence and having a retrospective design. Additionally, none of the studies was designed specifically to represent national population. Only well-designed studies with standard criteria can obtain accurate data for comparable studies on stroke, a disorder with several different pathologies and clinical presentations. Furthermore, the diverse society, heterogeneous ethnicity and marked geographical variation in the country[1] highlight the importance of conducting large studies, particularly in more populous parts in the north and the capital, to up-grade the current data to national figures. Retrospective collection of information is more liable to cause uncertainty and blurring of clinical differentiation between transient ischaemic attack and stroke; using a coding system is unreliable and vulnerable to diagnostic and administrative errors.

Since the general health care is divided between the private sector and general hospitals, the maintenance of contact, referral and follow up systems are lacking. There is no regular system of primary health care, neither is there regular contact with an organized nursing system to fulfil the sufficient criteria for adequate case ascertainment. Moreover, many patients with stroke in developing countries probably consult a private doctor before seeking hospital[42]. Repeatedly, a substantial minority presented to hospital are not admitted, highlighting the importance of the need for an expansion of case ascertainment to a comprehensive out-of-hospital case-registry system. Such a system should include general practitioners, private neurology sectors, private neuro-imaging centres and other potential sources for identifying stroke events, risk factors and outcomes. Documentation and record keeping is vital; so is more publication in internationally readable and accessible locations. By taking these measures, the current knowledge gap on stroke burden can be tackled in the country.

### Stroke patterns and risk factors

Likewise in developed countries, ischaemic stroke represents the majority of stroke subtypes, followed by primary intracranial haemorrhage and subarachnoid haemorrhage[10,20,34]. In regards to risk factors of ischaemic stroke, high blood pressure is the commonest risk factor, as expected, followed by cardiac causes, smoking and diabetes, respectively[10,17]. A survey of risk factors of non-communicable diseases representing Iranian adult population showed the prevalence of hypertension and the rate of self-awareness is unacceptably high, with 25% of population aged 25-64 years being hypertensive and an additional 46% being pre-hypertensive[43]. The study showed that only 34% of hypertensive patients were aware of their elevated blood pressure and 25% were taking anti-hypertensive medications[43]. The findings place the prevalence of hypertension, obesity and overweight in Iran as high as those in the United States, with Iranian women, in contrast with men, being more obese than their American counterpartners[44]. The highly prevalent risk factors pattern is similar to the other Arab countries neighbouring the Persian Gulf[45]. Unlike developed countries, rheumatic heart disease seems to be the most common cause of cardiac sources of embolism and many from this group are not adequately managed for secondary prevention of cardio-embolic stroke[16,17]. Otherwise stroke risk factors are similar to the developing countries.

A risk factor analysis study comparing the anterior and posterior circulation strokes showed that hypertension was a major risk factor for posterior circulation stroke, followed by smoking. Hyperlipidaemia, however, was equally increased in both anterior and posterior circulation strokes[46]. Although this study was not designed for epidemiological evaluation, its investigation showed some difference in comparison with the Canadian study[47]. Diabetes mellitus (DM) was related to an increased odds of posterior circulation ischemic stroke in the latter, compared with no higher prevalence of stroke associated with DM between anterior and posterior circulation strokes in Iran[46,47].

The Practical Iranian Criteria (PIC), which was designed for risk factor analysis and used for the largest epidemiological study of stroke pattern, was found reliable and useful in clinical practice[25]. It may seemingly be beneficial to standardize the criteria nationally, but lack of international recognition would limit the national studies for worldwide comparisons. Moreover, the PIC classification categorizes large artery atherosclerosis and microangiopathy as atherosclerotic mechanism, which results in an increased report of this subtype[17]. New measures and standardised risk factor analysis should also be included due to the substitution of modern life-style accompanied with higher health hazards, as well as increased global and local life-expectancy urges prevention programmes.

### Case fatality

The hospital-based 28-day case fatality rate in Iran is reported at 19.2% [10] and 31.5% [20]. The former had studied predominantly rural population near the Caspian Sea with reputation to eat seafood and have healthy diet, whereas the latter consists of over 94% urban population in the Centre of Iran. Interestingly, the incidence of stroke in the rural area at North is almost halved compared with the other urban study[20]. It may be concluded that lifestyle habits or local genetic population may explain the difference in incidence and case fatality between the two. Since there is no study designed to compare urban and rural data, the main reasons remain unclear and further studies are needed.

The case fatality of 31.5%, which was reported in one study, is comparable to the figures in Sub-Saharan Africa (30%)[33], China (27.2% for men & 32.9% for women)[37], Latin America & the Caribbean (19.3 to 26.2%)[36] and India (24.5% for urban and 37.1% for rural population)[35]. Although the case fatality is higher than Japan (17%), the figure is similar to the average of 22.9% reported from 13 countries from various parts of the world[34]. However, the relevance of the death registration system in Iran is highly suboptimal and the reported cause of stroke death could be up to 15% higher than the current report[48].

## Conclusion

Data on the epidemiology of stroke, its pattern and risk factors from Iran is scarce. However, there are numerous case series, mostly well-designed for the local community, reported in the literature highlighting relatively low incidence of stroke. This may reflect a similarity towards the neighbouring Arab nations, and a contrast with the West. It may however, support the lack in nationwide reliable data on the epidemiology of stroke and its risk factors, frequently seen in developing countries. Without an intervention, an increase in stroke burden is expected, as a result of ageing, population growth and "health transition"[49,50]. To enable better healthcare planning in Iran, there is therefore, a clear necessity for well-designed population-based comprehensive studies, updated and fulfilling published quality criteria, along with public education and awareness to fighting this disabling condition in this part of the world.

## Competing interests

The authors declare that they have no competing interests.

## Authors' contributions

AH designed the paper, carried out the literature search, data collection and selection (including official data from Iranian ministry of Health), analysis and interpretation of Persian and English data, and wrote the drafts. DSR participated in data collection and critical review of intellectual content. KG participated in critical review of intellectual content. HB conceived of the study, helped in the design of the paper, participated in analysis and interpretation of data and critical review of intellectual content. All authors read and approved the final manuscript.

## Pre-publication history

The pre-publication history for this paper can be accessed here:

http://www.biomedcentral.com/1471-2377/10/72/prepub
